# Supplementary material for: The Evolution and Characterization of the RNA Interference Pathways in Lophotrochozoa
Source: Genome Biol Evol. 2024 May 7;16(5):evae098. doi: 10.1093/gbe/evae098 (PMC11114477; doi:10.1093/gbe/evae098)
Supplement: evae098_Supplementary_Data [file evae098_supplementary_data.zip › Supplementary_materials.zip!/selected_miRNAs/NoGe_selected_miRNAs/mirdeep_pdf_files/NMRB01002591.1_Notospermus_geniculatus_strain_Ushimado_scaffold2591,_whole_genome_shotgun_sequence_39373.pdf]

Provisional ID	:	NMRB01002591.1_Notospermus_geniculatus_strain_Ushimado_scaffold2591,_whole_genome_shotgun_sequence_39373
Score total	:	1243.9
Score for star read(s)	:	3.9
Score for read counts	:	1234.9
Score for mfe	:	2.1
Score for randfold	:	0
Score for cons. seed	:	3
Total read count	:	2434
Mature read count	:	2425
Loop read count	:	0
Star read count	:	9

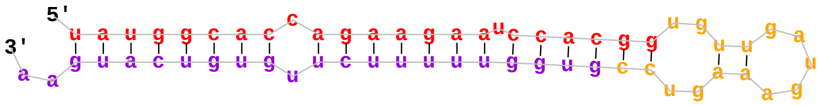

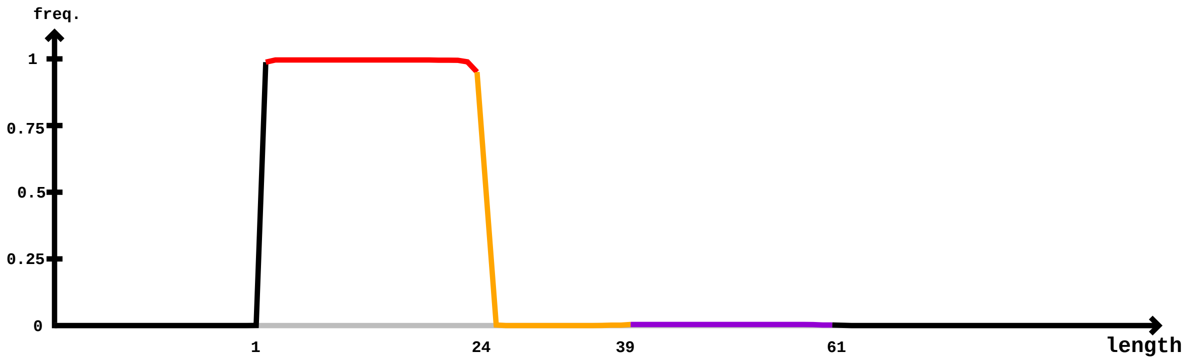

Mature Star

```
5'- uuugcuuuugucgugccauuuauggcaccagaagaauccacgguguugaugaaaguccgugguuuuucuugugucaugaauggucaugauuaggucgcuucgagugguuugug  -3'   obs
    uuugcuuuugucgugccauuuauggcaccagaagaauccacgguguugaugaaaguccgugguuuuucuugugucaugaauggucaugauuaggucgcuucgagugguuugug         exp
    ...((((..((((((((((((((((((.(((((((.((((((..((.....))..))))))))))))).)))))))))))))).)))))).))))((((...)))).......   reads   mm      sample
    ....................uauggcaccagaagaauccac........................................................................   2       0       Mm3
    ....................uauggcacUagaagaauccac........................................................................   1       1       Mm3
    ....................uauggcaccagaagaauccacg.......................................................................   8       0       Mm3
    ....................uauggcaccagaagaauccacgU......................................................................   2       1       Mm3
    ....................uauggcaccagaagaGuccacgg......................................................................   1       1       Mm3
    ....................uauggcaccagaagaauccacgA......................................................................   1       1       Mm3
    ....................uauggcaccagaagaauGcacgg......................................................................   2       1       Mm3
    ....................uauggcaccagaagaauccacgg......................................................................   181     0       Mm3
    ....................uauggcaccagaagaauUcacgg......................................................................   1       1       Mm3
    ....................uauggcacAagaagaauccacgg......................................................................   1       1       Mm3
    ....................uauggcaccagaagaaCccacgg......................................................................   1       1       Mm3
    ....................uauggcaccagUagaauccacgg......................................................................   1       1       Mm3
    ....................uauggcaccagaagaauccacggA.....................................................................   1       1       Mm3
    ....................uauggcaccagaagaauccacggu.....................................................................   10      0       Mm3
    .....................auggcaccagaagaauccacgg......................................................................   2       0       Mm3
    .....................auggcaccagaagaauccacggu.....................................................................   1       0       Mm3

    ....................uauggcaccagaagaauccac........................................................................   1       0       Fm3
    ....................uauggcacUagaagaauccac........................................................................   1       1       Fm3
    ....................uauggUaccagaagaauccacg.......................................................................   1       1       Fm3
    ....................uauggcaccagaagaauccacg.......................................................................   4       0       Fm3
    ....................uauggcaccagaagaaCccacgg......................................................................   1       1       Fm3
    ....................uauggcacUagaagaauccacgg......................................................................   2       1       Fm3
    ....................uauggcacGagaagaauccacgg......................................................................   1       1       Fm3
    ....................uauggcaccagaagaauccacgg......................................................................   21      0       Fm3

    ...................Cuauggcaccagaagaauccacgg......................................................................   1       1       Mm2
    ....................uauggcaccagaagaauc...........................................................................   2       0       Mm2
    ....................uauggcaccagaagaaucca.........................................................................   1       0       Mm2
    ....................uauggcacUagaagaauccac........................................................................   8       1       Mm2
    ....................uauggcaccagaagaauGcacg.......................................................................   1       1       Mm2
    ....................uaugUcaccagaagaauccacg.......................................................................   1       1       Mm2
    ....................uauggcaccagaagaauccacg.......................................................................   44      0       Mm2
```

Mature **Star**

```
uuugcuuuugucgugccauuuauggcaccagaagaauccacgguguugaugaaaguccgugguuuuucuugugucaugaauggucaugauuaggucgcuucgagugguuugug
....................uauggcaccagaagaGuccacgg......................................................................  1     1  Mm2
....................uauggcaccagaaUaauccacgg......................................................................  1     1  Mm2
....................uauggcaccagaagaauccacgA......................................................................  4     1  Mm2
....................uauggcacUagaagaauccacgg......................................................................  2     1  Mm2
....................uauggcaccagaagaauccacgU......................................................................  9     1  Mm2
....................uauggcaccagaagaauccacgg......................................................................  1482  0  Mm2
....................uauggcaccagaagaauUcacgg......................................................................  8     1  Mm2
....................uauggcaccagaaAaauccacgg......................................................................  1     1  Mm2
....................uauggcaccagaagaauGcacgg......................................................................  6     1  Mm2
....................uauggcaccagaagaaAccacgg......................................................................  4     1  Mm2
....................uauggcaccGgaagaauccacgg......................................................................  2     1  Mm2
....................uUuggcaccagaagaauccacgg......................................................................  1     1  Mm2
....................uauggcaccagaagaUuccacgg......................................................................  1     1  Mm2
....................uauggcaccagaGgaauccacgg......................................................................  5     1  Mm2
....................uauggcaccaAaagaauccacgg......................................................................  1     1  Mm2
....................uauggcaccagaagaauAcacgg......................................................................  20    1  Mm2
....................uauggcaccagaagaaGccacgg......................................................................  3     1  Mm2
....................uauggcaccagaagaauccacgC......................................................................  3     1  Mm2
....................uauggcaccagaagaauccaUgg......................................................................  1     1  Mm2
....................uauggcaccagaUgaauccacgg......................................................................  1     1  Mm2
....................uauggcaccagaagaaucUacgg......................................................................  1     1  Mm2
....................Nauggcaccagaagaauccacgg......................................................................  1     1  Mm2
....................uauggcaccagaagaaCccacgg......................................................................  3     1  Mm2
....................uauggcaccagaagaauccacggA.....................................................................  1     1  Mm2
....................uauggcaccGgaagaauccacggu.....................................................................  1     1  Mm2
....................uauggcaccagaagaauccacggu.....................................................................  40    0  Mm2
....................uauggcaccagaagaauAcacggu.....................................................................  2     1  Mm2
....................uauggcaccagaagaauccacgguU....................................................................  4     1  Mm2
.....................auggcaccagaagaauccacg.......................................................................  1     0  Mm2
.....................auggcaccagaagaauccacgg......................................................................  6     0  Mm2
.....................auggcaccagaagaauccacggu.....................................................................  1     0  Mm2
.......................................................uccgugguuuuucuugugucau....................................  1     0  Mm2
..........................................................gugguuuuucuugugucaug...................................  1     0  Mm2
..........................................................gugguuuuucuugugucaugaa.................................  2     0  Mm2

....................uauggcaccagaagaauccacg.......................................................................  32    0  Mm1
....................uauggcaccagaagaaGccacgg......................................................................  1     1  Mm1
....................uauggcaccagaagaauccacgA......................................................................  6     1  Mm1
....................uauggcaccaAaagaauccacgg......................................................................  1     1  Mm1
....................uauggcaccagaagaauccacgg......................................................................  212   0  Mm1
....................uUuggcaccagaagaauccacgg......................................................................  1     1  Mm1
....................uauggcaccagaagaauAcacgg......................................................................  2     1  Mm1
....................uaugAcaccagaagaauccacgg......................................................................  1     1  Mm1
....................uauggcaccagaagaauGcacgg......................................................................  1     1  Mm1
....................uauggcaccagaagaaAccacgg......................................................................  1     1  Mm1
....................uauggcaccagaagaauccacggA.....................................................................  6     1  Mm1
....................uauggcaccagaagaauccacggu.....................................................................  9     0  Mm1
.....................auggcaccagaagaauccacgg......................................................................  8     0  Mm1
..........................................................gugguuuuucuugugucaugaa.................................  3     0  Mm1

....................uauggcaccagaagaauccacg.......................................................................  1     0  Fm1
....................uauggcaccagaagaauAcacgg......................................................................  1     1  Fm1
....................uauggcaccagaagaauccacgg......................................................................  118   0  Fm1
....................Nauggcaccagaagaauccacgg......................................................................  1     1  Fm1
....................uauggcaccagaagaauccacUg......................................................................  1     1  Fm1
....................uauggcaccagaagaauUcacgg......................................................................  1     1  Fm1
....................uauggcaccagaagaauccacgU......................................................................  2     1  Fm1
.....................auggcaccagaagaauccacgg......................................................................  1     0  Fm1
........................................................ccgugguuuuucuugugucaug...................................  3     0  Fm1

....................uauggcacUagaagaauccac........................................................................  1     1  Fm2
....................uauggcaccagaagaaAccacgg......................................................................  1     1  Fm2
....................uauggcaccagaagaauccacgg......................................................................  82    0  Fm2
....................uauggcaccaCaagaauccacgg......................................................................  1     1  Fm2
....................uauggcaccagaagaauGcacgg......................................................................  2     1  Fm2
....................uauggcaccagaagaauAcacgg......................................................................  1     1  Fm2
....................uauggcaccagaagaauccacggu.....................................................................  6     0  Fm2
....................uauggcaccagaagaauccacgguA....................................................................  1     1  Fm2
```
